# The role of miRNA in diagnosing and clarifying the pathomechanisms in major depressive disorder

**DOI:** 10.1192/j.eurpsy.2023.762

**Published:** 2023-07-19

**Authors:** C. S. Ho, M. W. Soh, G. Tay

**Affiliations:** Psychological Medicine, National Univeristy of Singapore, Singapore

## Abstract

**Introduction:**

There are currently no diagnostic or treatment-guiding biomarkers for major depressive disorder (MDD). Microribonucleic acids (miRNA) may facilitate understanding the reorganisation of gene expression networks in MDD. Identifying miRNA and target mRNA pathways that contribute to MDD may open new therapeutic avenues, such as inhibiting endogenous miRNA or administering exogenous miRNA.

**Objectives:**

This study investigates how miRNAs can clarify the molecular mechanisms of MDD by comparing the miRNA levels in the blood serum of patients with MDD and healthy individuals. The study also investigates the discriminative ability of miRNAs to distinguish between depressed patients and healthy controls.

**Methods:**

Sixty depressed patients were matched with 60 healthy controls based on age, gender, ethnicity, and years of education. The severity of depression was measured using the Hamilton Depression Rating Scale, and venous blood was collected for miRNA profiling. Using the QIAGEN Ingenuity Pathway Analysis, networks were constructed to identify the biological pathways associated with MDD influenced by the differentially expressed miRNAs. Analyses of the receiver operating characteristic (ROC) were performed to examine the capacity of miRNAs to distinguish between depressed and healthy individuals.

**Results:**

Six miRNAs (miR-542-3p, miR-181b-3p, miR190a-5p, miR-33a-3p, miR-3690, and miR-6895-3p) were significantly down-regulated in untreated depressed patients compared to healthy controls. miR-542-3p has experimentally validated mRNA targets predicted to be associated with MDD. ROC analyses determined that a panel containing miR-542-3p, miR181b-3p, and miR-3690 distinguished between depressed and healthy individuals with an area under the curve value of 0.67.

**Conclusions:**

Specific miRNAs, including miR-542-3p, miR181b-3p, and miR-3690, may be biomarkers with targets implicated in the pathophysiology of depression. They could also be used to distinguish accurately between depressed and healthy individuals.

**Disclosure of Interest:**

None Declared

